# Association between obesity indicators and cardiometabolic disease in Chinese adults

**DOI:** 10.1371/journal.pone.0273235

**Published:** 2023-01-20

**Authors:** Jiang Wu, Li Zou, Yin Liu, Hanbing Yu, Hua Yin, Lisheng Zhong, Yifang Liu, Wenning Fu, Shengchao Zhang

**Affiliations:** 1 General Practice Department, Baoan Central Hospital of Shenzhen, Shenzhen, Guangzhou, China; 2 Department of Neurology, Zhongnan Hospital of Wuhan University, Wuhan, China; 3 Office of Basic Public Health, Baoan Central Hospital of Shenzhen, Shenzhen, Guangzhou, China; 4 Fuzhongfu Community Health Service Center, Baoan Central Hospital of Shenzhen, Shenzhen, Guangzhou, China; 5 Yikangyuan Community Health Service Center, Baoan Central Hospital of Shenzhen, Shenzhen, Guangzhou, China; 6 Yantian Community Health Service Center, Baoan Central Hospital of Shenzhen, Shenzhen, Guangzhou, China; 7 School of Nursing, Tongji Medical College, Huazhong University of Science and Technology, Wuhan, Hubei, China; 8 Key Laboratory of Emergency and Trauma, Ministry of Education, College of Emergency and Trauma, Hainan Medical University, Haikou, Hainan, China; Università degli Studi di Milano, ITALY

## Abstract

**Background:**

Obesity is an established risk factor for cardiometabolic disease. Different measurements of obesity with cardiometabolic disease have been compared in recent studies in Western countries. However, obesity-related criteria for the Chinese population differ from the standard World Health Organization guidelines, and similar research in Chinese adults is limited.

**Measures:**

Data were obtained from a comprehensive intervention project involving a community population with cardiovascular and cerebrovascular risk factors in Shenzhen in 2015. A total of 4,000 participants (1,605 men and 2,395 women) with a mean age of 56.01±9.78 years were included in this study. Categorical data are reported as percentages, and continuous data are reported as mean ± standard deviation. Logistic regression analyses were conducted to examine the associations of body mass index (BMI), waist circumference (WC), and neck circumference (NC) with hypertension, diabetes, and dyslipidemia among Chinese adults.

**Results:**

The participants had a mean BMI of 24.25±3.33 kg/m^2^, mean NC of 33.59±4.16 cm, and mean WC of 82.44±9.84 cm (men: 85.46±9.10 cm, women: 80.40±9.81 cm). Blood pressure, plasma glucose, and lipid levels in the BMI, WC, and NC groups were statistically significant (*p* < 0.05). BMI, WC, and NC were positively correlated with systolic blood pressure, diastolic blood pressure, fasting plasma glucose, total cholesterol, and triglyceride and negatively correlated with low-density lipoprotein cholesterol (*p* < 0.05), while the risk of hypertension, diabetes, and dyslipidemia increased with an increase in BMI, WC, and NC (*p* < 0.05). One SD of BMI, WC, and NC resulted in an increase of 41%, 22%, and 31% risk of hypertension; 45%, 34%, and 47% risk of diabetes; and 37%, 32%, and 23% risk of dyslipidemia, respectively.

**Conclusions:**

Compared to BMI and NC, WC was more strongly associated with cardiometabolic diseases in Chinese adults.

## Introduction

Cardiometabolic diseases, such as diabetes, dyslipidemia, and cardiovascular disease [1], are the most important causes of morbidity and mortality worldwide, and their prevalence has increased substantially in recent years, imposing a heavy burden on the health care system [1, 2]. Obesity is a vital risk factor for cardiometabolic diseases [3], and its harm has been gradually understood by the majority of people. Related studies have emphasized that selecting reasonable obesity indicators is beneficial for preventing and controlling cardiometabolic diseases [4]. Previous studies have reported that body mass index (BMI), a measure of adiposity, is a predictor of cardiometabolic diseases. However, it can only reflect the total weight of the body [5], not the distribution of body fat or the proportion of fat in body composition. Subsequently, a growing amount of evidence has indicated that waist circumference (WC) is also related to cardiometabolic disease; however, it could not accurately reflect visceral fat related to metabolic disorders [6]. Neck circumference (NC) is the circumference of the laryngeal node and can effectively reflect the subcutaneous fat in the neck or the deposition of fat around the respiratory tract. It is an important indicator of fat distribution in the upper body [7]. Recent studies have shown that NC is related to hypertension, diabetes, and dyslipidemia [8–11]. Although scholars have studied the relationship between BMI, WC, NC, hypertension, diabetes, and dyslipidemia, the obesity index can better reflect the impact of obesity on hypertension, diabetes, and dyslipidemia; however, the relevant research results are still inconsistent [12–14]. Moreover, there are significant differences in the genetic backgrounds and BMI criteria for obesity between Asian and Western populations [15]. China is the most populous nation in the world, with one-fifth of the world’s population, and similar research in China is scarce; the related research results among the general Chinese population are controversial. Therefore, the present study aimed to compare BMI, WC, and NC, which have a strong relationship with hypertension, diabetes, and dyslipidemia, in Chinese adults.

## Subjects and methods

### Ethics statement

The study was approved by the Research Ethics Committee in Baoan Central Hospital of Shenzhen, China. All participants read a statement that explained the purpose of the survey and provided written informed consent before participating in the study.

### Study population and sampling

This cross-sectional survey was conducted between January 2015 and March 2016 in Xixiang Street, Bao’an District, Shenzhen (southeast China). Xixiang Street has 33 communities. Taoyuan Ju, Liutang, and Xixiang were selected for our study, which satisfied the following eligibility criteria: (1) has medical staff working in community health service centers who actively participate in health education programs; (2) provides a well-maintained health record; and (3) is a national comprehensive prevention and treatment zone for chronic diseases or a national monitoring point for chronic diseases. Subsequently, the community was surveyed using a multi-stage stratified cluster survey; in the first phase of sampling, a computer program was used to randomly select 4202 households in three selected communities with the following requirements: (1) participants had a permanent residence for a minimum of 6 months annually in the community to ensure they could be contacted and age was restricted to 40 years and above; (2) participants with mental disorders were prohibited from participating; and (3) participants expressed consent to participate. Questionnaire completion and assessment of cardiovascular disease risk factors were conducted in community health centers. All adults were selected for further assessment of blood pressure and physical examination. All eligible participants were registered at the local government and were informed to undergo physical and laboratory examinations in community health centers at a specific time. Participants were allowed to obtain the results from community health centers 2 weeks later.

### Date collection

Trained medical staff asked the participants to provide information regarding their cardiovascular disease history and risk factors. All data were collected by a neurologist or trained physician through a structured questionnaire upon exit from the community health center to obtain detailed information, including demographic data, cardiovascular disease history, diagnosis date, lifestyle risk factors, chronic diseases, and status of risk factors. The senior investigators assessed the collected questionnaires daily as a quality control measure. The data were entered into the database in a double-blind manner by two different researchers using EpiData 3.0 to guarantee accuracy. The physical examination included measurements of height, weight, NC, and WC. Blood samples were obtained after an overnight fast to examine the participants’ biochemical characteristics. Individuals were excluded if they had been diagnosed with an illness or malignant disease. After exclusion of missing data and extreme values, 4,000 participants were included in the analyses.

### Anthropometric and biomarker measurements

NC was measured with the head erect and eyes facing forward at the upper margin of the laryngeal prominence. A portable stadiometer was used to measure the height without shoes to the nearest 0.1 cm. Weight in light clothing was measured to the nearest 0.1 kg using a digital scale. WC was measured to the nearest 0.1 cm at the midpoint between the iliac crest and lower rib.

All participants provided blood samples after a >8-h overnight fast, which were collected in glass tubes and allowed to clot at room temperature. Total cholesterol (TC), triglyceride (TG), low-density lipoprotein cholesterol (LDL-C), high-density lipoprotein cholesterol (HDL-C), fasting plasma glucose (FPG), and 2-h post-load plasma glucose (2hPG) levels were measured using an enzymatic calorimetric test at the local hospital. Although global clinical practice guidelines recommend the use of the Friedewald formula (FF) to calculate LDL-C levels in routine patients [16], the enzymatic method was chosen for this study to measure LDL-C, considering that FF tends to underestimate LDL-C levels in the presence of high TG levels [17].

### Blood pressure measurements and assessment of related disease

After the individuals had rested in a chair with back support for 10 min, their blood pressure was measured. A trained nurse measured blood pressure using an automated sphygmomanometer. Both feet were placed flat on the floor, and the arms were supported at the heart level. Three measurements were taken at 5-min intervals during 8–9 AM, and the mean value was recorded for the analyses. Before the evaluation, the participants were instructed to refrain from drinking alcohol, tea, or coffee and from smoking or exercising for ≥30 min.

Hypertension was defined as a systolic blood pressure (SBP) ≥140 mmHg, diastolic blood pressure (DBP) ≥90 mmHg, or current use of antihypertensive medication [18]. Diabetes was defined as an FPG value ≥7.0 mmol/L, a 2hPG value ≥11.1 mmol/L, or current treatment with insulin or other hypoglycemic agents [19]. Dyslipidemia was defined as follows: TC ≥6.22 mmol/L, or HDL-C <1.04 mmol /L, or LDL-C ≥4.14 mmol /L, or TG ≥2.26 mmol /L [20].

### Statistical methods

We examined kurtosis and skewness (skewness ≤2; kurtosis ≤7) to test the normality distribution of the variables. The results showed that the data were normally distributed [21]. All categorical data are reported as numbers and percentages, whereas continuous data are reported as mean ± standard deviation (SD). The Levene’s test verified the homogeneity of variances, thus the differences in the distribution of continuous variables between men and women were tested using Student’s t-test. The chi-square test for categorical variables. Logistic regression models were used to evaluate the associations between BMI, WC, NC, hypertension, diabetes, and dyslipidemia. In the logistic regression analysis, covariates included age (continuous year), education (primary school, junior high school, high school, junior college, master’s degree), smoking (yes or no), drinking (yes or no), and physical activity (yes or no). All statistical analyses were performed using SPSS 19.0 (SPSS Inc., Chicago, Ill, USA). Differences were considered statistically significant at *p* < 0.05.

## Results

Of the 4000 individuals who were included in the final analysis, there were 1606 men (mean age, 56.02±10.15 years) and 2395 women (mean age, 55.59±9.53 years). Table 1 shows the demographics, lifestyle, and anthropometric characteristics of the overall sample, as well as the gender of the participants. Participants had a mean BMI of 24.25±3.33 kg/m^2^ (men: 24.53±3.07 kg/m^2^, women: 24.06±3.48 kg/m^2^), mean NC of 33.59±4.16 cm (men: 33.50±4.23 cm, women 33.32±3.59 cm), and mean WC of 82.44±9.84 cm (men: 85.46±9.10 cm, women: 80.40±9.81 cm). Regarding educational level, most participants finished primary school accounting for 33.2%. Compared with women, men were observed to have a significantly higher SBP, DBP, and FPG, and men were more likely to be smokers and drinkers and physically active (all *p* < 0.05).

**Table 1:** Characteristics of the study participants stratified by gender.

Variable	Total (*n* = 4000)	Men (*n* = 1605)	Women (*n* = 2395)	*P*
Age(years)	56.01±9.78	56.02±10.15	55.95±9.53	<0.001
BMI (kg/m^2^)	24.25±3.33	24.53±3.07	24.06±3.48	0.014
NC (cm)	33.59±4.16	35.50±4.23	32.32±3.59	<0.001
WC (cm)	82.44±9.84	85.46±9.10	80.42±9.81	0.048
SBP (mmHg)	126.16±18.48	128.03±17.31	124.90±19.13	<0.001
DBP (mmHg)	82.08±11.55	84.66±11.74	80.37±11.09	0.026
FPG (mmol/L)	5.74±6.43	5.97±10.02	5.60±1.44	0.027
Education				
Primary school	1331(33.2)	327 (20.4)	1004 (42.0)	0.040
Junior high school	1206(30.2)	502 (31.3)	704 (29.4)	
High school	910(22.8)	437 (27.1)	473 (19.7)	
Junior College	545(13.6)	333 (20.7)	212 (8.8)	
Master degree	8(0.2)	6 (0.4)	2 (0.08)	
Smoking [n (%)]	592(14.8)	570 (35.6)	22 (0.9)	<0.001
Drinking [n (%)]	571(14.3)	482 (30.1)	89 (3.7)	<0.001
Physical activities [n (%)]	161(4.0)	91(5.7)	70 (2.9)	<0.001

Categorical data were reported as percentages and continuous data were reported as means ±standard deviation.

Abbreviations BMI: body mass index; WC: waist circumference; NC: neck circumference; SBP: systolic blood pressure; DBP: diastolic blood pressure. FPG: Fasting plasma glucose. Fasting plasma glucose 2hPG:2-hour post-load plasma glucose. TG: Triglycerides; TC: Total cholesterol; LDL-C: Low-density lipoprotein; HDL-C: High-density lipoprotein.

Blood pressure, blood glucose, and blood lipid levels between the NC, BMI, and WC groups are shown in Table 2. Blood pressure, plasma glucose, and lipid levels in the BMI, WC, and NC groups were significantly different (*p* < 0.05). BMI, WC, and NC were positively correlated with SBP, DBP, FPG, TC, and TG and negatively correlated with LDL-C (*p* < 0.05).

**Table 2:** The distribution of blood pressure, plasma glucose and blood lipid levels among different BMI and WC (Mean ± SD).

Variables	SBP	DBP	FPG	TG	TC	LDL-C	HDL-C
**BMI (kg /m** ^**2**^ **)**							
<22.10	121.63±18.21	79.08±10.94	5.84±12.64	1.42±1.21	4.79±1.01	3.37±1.13	0.92±0.43
22.10–24.12	125.33±18.23	81.16±11.02	5.61±1.44	1.66±1.30	4.93±0.92	3.52±1.12	0.89±0.37
24.12–26.14	127.55±18.23	82.74±11.86	5.71±1.49	1.72±1.31	4.90±1.08	3.58±1.22	0.90±0.89
>26.14	130.23±18.22	85.38±11.37	5.81±1.33	2.05±1.51	5.01±1.72	3.75±1.22	0.89±0.57
**WC (cm)**							
<77	121.80±18.73	79.64±11.45	5.37±1.44	1.41±1.20	4.84±0.96	3.46±1.21	0.92±0.42
77–82	125.13±17.80	81.04±10.68	5.55±1.26	1.65±1.34	4.96±1.10	3.52±1.13	0.94±0.89
82–89	127.19±18.01	82.41±11.40	6.07±12.27	1.78±1.38	4.88±0.99	3.60±1.16	0.89±0.62
>89	131.53±17.96	85.84±11.66	6.03±1.67	2.07±1.45	4.97±1.78	3.68±1.21	0.86±0.36
**NC (cm)**							
<31	123.13±12.89	78.97±11.18	5.41±1.28	1.48±1.34	4.87±1.07	3.40±1.14	0.95±0.45
31–33	124.36±18.96	80.87±10.59	5.61±1.48	1.64±1.18	4.94±1.06	3.50±1.16	0.90±0.52
33–36	128.91±18.22	84.05±11.05	6.09±12.31	1.78±1.42	4.93±1.64	3.66±1.20	0.89±0.88
>36	129.46±17.98	85.70±12.29	5.95±1.61	2.06±1.44	4.87±0.95	3.73±1.21	0.84±0.41

BMI: body mass index; WC: Waist circumference; NC: Neck circumference; SBP: Systolic blood pressure; DBP: Diastolic blood pressure; FPG: fasting plasma glucose; TG: triglyceride; TC: total cholesterol; LDL-C: low density lipoprotein-cholesterol; HDL-C: high density lipoprotein-cholesterol.

Associations between BMI, WC, and NC with hypertension, diabetes, and dyslipidemia are shown in Table 3. Logistic regression analysis was performed for hypertension, diabetes, and dyslipidemia (dependent variables), and BMI, WC, and NC (independent variables) were adjusted for gender, age, educational level, smoking status, alcohol consumption, and physical exercise. The results showed that the risks of hypertension, diabetes, and dyslipidemia increased with increases in BMI, WC, and NC. The risks of hypertension, diabetes, and dyslipidemia in the highest quartile of BMI were 3.11 (odds ratio [OR] = 3.11, 95% confidence interval [CI]: 2 47–3.92), 1.68 (OR = 1.68, 95% CI: 1.22–2.31), and 3.28 (OR = 3.28, 95% CI: 2 76–3.88) times than the lowest quartile group, respectively. The risk of the three chronic diseases in the highest quartile of WC was 2.66 (OR = 2.66, 95% CI: 2.12–3.42), 2.10 (OR = 2.10, 95% CI: 1.52–2.91), and 3.76 (OR = 3.76, 95% CI: 3.15–4.18) times that in the lowest quartile group, respectively. The risk of the three chronic diseases in the highest quartile of NC was 2.55 (OR = 2.55, 95% CI: 1.97–3.29), 2.95 (OR = 2.95, 95% CI: 2.05–4.23), and 3.09 (OR = 3.09, 95% CI: 2.62–3.65) times that in the lowest quartile group, respectively (Table 3).

**Table 3:** The relationship between BMI, WC, NC and the prevalence of new hypertension, diabetes and dyslipidemia.

Variables	hypertension		diabetes		dyslipidemia	
	OR	95% CI	OR	95% CI	OR	95% CI
**BMI (kg/m** ^**2**^ **)**						
<22.10	1.00			1.00		1.00
22.10–24.12	1.81	1.42–2.30	1.29	0.93–1.80	1.36	1.14–1.62
24.12–26.14	2.04	1.61–2.59	1.27	0.91–1.77	2.00	1.68–2.38
>26.14	3.11	2.47–3.92	1.68	1.22–2.31	3.28	2.76–3.88
**WC (cm)**						
<77	1.00			1.00		1.00
77–82	1.33	1.05–1.68	1.20	0.85–1.69	1.52	1.26–1.82
82–89	1.66	1.32–2.07	1.42	1.03–1.98	2.13	1.78–2.55
>89	2.66	2.12–3.42	2.10	1.52–2.91	3.76	3.15–41.8
**NC (cm)**						
<31	1.00			1.00		1.00
31–33	1.51	1.20–1.88	1.43	1.03–1.99	1.63	1.02–2.63
33–36	2.02	1.62–2.52	1.67	1.20–2.31	2.66	1.71–1.79
>36	2.55	1.97–3.29	2.95	2.05–4.23	3.09	2.62–3.65

BMI: body mass index; WC: Waist circumference; NC: Neck circumference; OR: Odds Ratio; CI: Confidence Interval.

Further analysis of the effects of the change for every SD of BMI, WC, and NC on the risk of hypertension, diabetes, and dyslipidemia was conducted, and the results suggested that the change for each SD of BMI, WC, and NC increased the risk of hypertension by 41% (OR = 1.41, 95% CI: 1.31–1.53), 22% (OR = 1.22, 95% CI: 1.10–1.36), and 31% (OR = 1.31, 95% CI: 1.32–1.51); increased the risk of diabetes by 45% (OR = 1.45, 95% CI: 1.34–1.56), 34% (OR = 1.34, 95% CI: 1.20–1.50), and 47% (OR = 1.47, 95% CI: 1.37–1.58); and increased the risk of dyslipidemia by 37% (OR = 1.37, 95% CI: 1.25–1.50), 32% (OR = 1.32, 95% CI: 1.18–1.48), and 23% (OR = 1.23, 95% CI: 1.16–1.51), respectively. The effect of WC on the risk of the three chronic diseases was higher than that of NC and BMI, as shown in Table 4.

**Table 4:** Association of BMI, WC and NC every change of 1 SD with the prevalence of hypertension, diabetes and dyslipidemia.

Indexes	SD	hypertension		diabetes		dyslipidemia	
		*β*	*OR* (95% *CI*)	*β*	*OR* (95% *CI*)	*β*	*OR* (95% *CI*)
BMI	3.33	0.35	1.41(1.31–1.53)	0.20	1.22(1.10–1.36)	0.38	1.31(1.32–1.51)
WC	9.29	0.37	1.45(1.34–1.56)	0.29	1.34(1.20–1.50)	0.41	1.47(1.37–1.58)
NC	3.64	0.31	1.37(1.25–1.50)	0.28	1.32(1.18–1.48)	0.29	1.23(1.16–1.51)

BMI: body mass index; WC: Waist circumference;NC: Neck circumference; OR: Odds Ratio; CI: Confidence Interval.

## Discussion

The rising prevalence of obesity in several countries has been described as a global pandemic [22–26]. In 2015, overweight and obesity were estimated to cause 3.4 million deaths and account for 4% of years of life lost and 4% of disability-adjusted life-years worldwide [27–29]. Data from studies in the USA have suggested that an increase in obesity could lead to future falls in life expectancy [30]. Obesity is closely related to the occurrence and death of chronic diseases such as hypertension, diabetes, and dyslipidemia, is considered to be one of the most important risk factors, and has become a major public health problem that threatens the health of people [31–33]. The results of this study indicated that with an increase in BMI, WC, and NC, the SBP, DBP, FPG, TC, TG, and LDL-C levels increased and HDL-C levels decreased, which is consistent with previous studies [34–36]. Some studies have found that leptin levels were higher in the obese population than in the normal population, and the opposite results were found for the adiponectin levels. Leptin activates the renal sympathetic nerve and elevates arterial blood pressure, and adiponectin protects the heart and blood vessels and shows a negative correlation with insulin resistance [37, 38]. Obesity can cause pancreatic insufficiency and reduce insulin secretion as fat cells are not sensitive to insulin, resulting in hyperglycemia [4]. The fat decomposition rate of visceral fat cells was higher in the obese population, and a large amount of free fatty acids were produced in the liver through the portal system, which increased the synthesis of triglycerides, and excessive deposition of fat could change the activity of lipoprotein lipase and accelerate the synthesis of cholesterol [39, 40].

BMI, WC, and NC are measurement indicators that reflect obesity levels from different perspectives. Moreover, previous studies have shown that [41, 42], in the prediction of obesity-related chronic diseases, WC was better than BMI and NC. Our study showed that for each SD increase in BMI, WC, and NC, the risk of hypertension increased by 41%, 45%, and 31%; the risk of diabetes increased by 22%, 34%, and 32%; and the risk of dyslipidemia increased by 31%, 47%, and 23%, respectively, indicating that regardless of BMI, WC, or NC, the risk of hypertension, diabetes, and dyslipidemia will increase, and WC has a greater impact on the risk of chronic diseases. Consistent with a statement from the American Heart Association (AHA) in 2015, the assessment method used to assess adiposity in Asian populations should be chosen carefully. Although BMI has been used to measure adiposity in most human races, including Asians, its sensitivity is low in assessing the risk of cardiovascular disease [43]. Instead, it has been suggested that WC should be used to assess the risk of cardiovascular diseases in Asians [33]. These findings could be explained by the fact that the mean or median BMI of Asian populations is lower than the current WHO classification of 25 kg/m as defining overweight. In addition, Chinese people tend to accumulate fat in the abdominal region of the body [44]. This may explain the stronger correlation between WC and cardiometabolic disease in Chinese adults. Excessive deposition of abdominal fat is found in the central obese population, which seriously affects the metabolism of glycolipids and increases the risk of chronic diseases [4, 37, 38]. According to the latest European Society of Cardiology/European Society of Hypertension guidelines, important risk factors for cardiometabolic disease include physical inactivity in addition to obesity [45]. It is recommended to reduce WC by appropriate physical exercise based on weight control to reduce the risk of obesity-related chronic diseases.

The present study has several strengths. First, this study is the first in China to compare the relationship of BMI, WC, and NC with hypertension, diabetes, and dyslipidemia. Additionally, the results of our study indicated that a stronger relationship was found for the effect of WC on hypertension, diabetes, and dyslipidemia, which was consistent with the results of a study conducted in 2015 in the USA, indicating that Asians should pay attention to using WC to assess cardiovascular and metabolic disease risks.

There are some limitations of the present study that should be considered. First, the cross-sectional setting limited its ability to infer causality from these results. Second, information about cardiovascular disease history and risk factor status was based on self reports. An Italian longitudinal study showed that low awareness of risk factors for cardiovascular disease among non-medical individuals and a lack of awareness of hypertension and dyslipidemia were positively associated with higher risk for atherosclerosis [46]. Although trained medical personnel conducted structured questioning of patients, the accuracy of the information could not be assumed, and recall bias could not be excluded. Third, since this study was conducted in 2015, the definitions of hypertension and dyslipidemia were based on clinical guidelines at that time. Considering that some guidelines have been updated with the development of modern medicine, this limitation should be carefully considered when generalizing the findings of this study.

## Conclusion

The results of the present study indicate that BMI, WC, and NC are simple and easy indicators of obesity and have important predictive values for the risk of cardiometabolic diseases, and the impact of WC had a greater predictive value. Thus, based on the evidence from the present study and the AHA statement in 2015, we suggest that in addition to BMI, WC should be documented for the assessment of the risk of cardiometabolic diseases in Chinese populations. Further prospective population-based and mechanistic studies are needed to validate our findings.

## Supporting information

S1 Data Minimal data set. (SAV)
